# Association Between *Helicobacter pylori* cagA, babA2 Virulence Factors and Gastric Mucosal Interleukin-33 mRNA Expression and Clinical Outcomes in Dyspeptic Patients

**Published:** 2015

**Authors:** Heshmat Shahi, Somayeh Reiisi, Rasol Bahreini, Nader Bagheri, Loghman Salimzadeh, Hedayatollah Shirzad

**Affiliations:** 1 *Cellular and Molecular Research Center, Shahrekord University of Medical Sciences, Shahrekord, Iran.*; 2 *Department of Genetic, Faculty of basic science, Shahrekoed University, Shahrekord, Iran.*; 3 *Department of Internal Medicine, Shahrekord University of Medical Sciences, Shahrekord, Iran.*

**Keywords:** *Helicobacter pylori*, gastritis, interleukin-33, virulence factor

## Abstract

*Helicobacter pylori* (*H. pylori*) infection has been reported in more than half of the world human population. It is associated with gastric inflammation and noticeable infiltration of the immune cells to the stomach mucosa by several cytokines secretion. IL-1β, IL-18 have been shown to contribute to *H. pylori* induced gastritis, but the details of inflammation and association of virulence factors remain unclear. IL-1 cytokine family has a new additional cytokine, Interleukin-33 (IL-33), which is contemplated to have an important role for host defense against microorganisms. *H. pylori* virulence factors important in gastritis risk are the cag pathogenicity island (cag-PAI) and babA. This study evaluated IL-33 mucosal mRNA expression levels in infected and uninfected patients and its relationship with bacterial virulence factors cagA, babA_2_ and type of gastritis. Total RNA was extracted from gastric biopsies of 79 *H. pylori*-infected patients and 51 *H. pylori*-negative patients. Mucosal IL-33 mRNA expression levels in gastric biopsies were assessed using real-time PCR. Existence of virulence factors were detected by PCR. IL-33 mRNA expression was significantly higher in biopsies of *H. pylori*-infected patients compared to *H. pylori*-uninfected patients (P<0.0001). Also there was a direct relationship between virulence factor bab-A_2_ and enhancement in IL-33 mRNA expression. Furthermore, IL-33 mRNA expression level was significantly lower in chronic gastritis patients compared with patients with active gastritis (P<0.001). IL-33 may play a crucial role in the inflammatory response and induction of the chronic gastritis and severity of inflammatory changes in the gastric mucosa.

*Helicobacter pylori* (*H. pylori*) is a Gram-negative bacterium that attaches to the stomach epithelial cells and colonizes in the stomach of approximately half of the world population, causing long lived gastric inflammation despite the host immune responses. (1, 2). *H. pylori* infection is associated with gastritis and immune cell infiltration into the gastric mucosa that may lead to chronic gastritis, peptic ulcer and production of pro inflammatory cytokines such as IL-1, IL-8, and TNFα (3, 4). In recent years, a newly addition to the IL-1 family is IL-33 which has high similarity to IL-18. In contrast to IL-18, IL-33 has anti-inflammatory effects although a TH1 response has been reported (5, 6). IL-33 is produced as a 30 kD protein which is cleaved by caspase to produce an 18 kD form. IL-33 is highly expressed in the stomach epithelium. However, its gastric function is unknown (5). IL-33 exists in the nucleus and cytoplasm of macrophages, dendritic cells, fibroblast and endothelial cells (7). The role of IL-33 and its receptors ST2 "IL-33/ST2" have been identified as crucial to the homeostasis of the epithelial inflammation specially in intestinal epithelium (8). Patients with ulcerative colitis show increased IL-33 levels in the epithelium of intestine (9). The ST2 gene relates to the IL-1/TLRs super family which produces four ST2 protein isoforms (10). In addition, serum ST2 levels are associated with severity of disease that may be a biomarker of disease degree (7, 11). Some researchers showed high expression level of IL-33 in endothelial cells and cancer cell lines (8, 12). IL-33 is known to be associated with inflammatory tissue in crohn’s disease and rheumatoid arthritis (9). Although virulence factors may induce gastric inflammation, atrophy, metaplasia and malignancy in the stomach, it has been reported that mucosal levels of pro-inflammatory cytokines in the infection site with *H. pylori* are correlated with the various *H. pylori* virulence factors (13). This study evaluated IL-33 mucosal mRNA expression levels in infected and uninfected patients and assessed its relationship with bacterial virulence factors cagA, babA_2_ and type of gastritis.

## Material & methods

**Patients & sampling**

130 specimens were collected from patients presenting dyspepsia symptoms and gastrointestinal disorders. The process was approved by Ethics Committee of Shahrekord University of Medical Sciences. Gastric biopsy specimens were taken from the antrum (pyloric gland area). Gastritis was investigated by endoscopy. None of the patients had received anticoagulants and nonsteroidal anti- inflammatory drugs (NSAIDs) for 1 month before specimen collection and none of them had received treatment for *H. pylori* infection and no autoimmune disease was reported (14). 79 *H. pylori* infected gastritis patients including 40 men (40.02 ± 15.65 years) and 39 women (38.9 ± 13 years) and 51 uninfected gastritis patients, 23 men (41.42 ± 12.25 years) and 28 women (39.8 ± 15.02 years), contributed to this study. *H. pylori* infection was detected by the rapid urease test (RUT), polymerase chain reaction (PCR), and pathological examination (PE) of three biopsies taken from the antrum. Patients with all positive tests (RUT, PCR, PE) were considered as positive for *H. pylori* infection. Determination of bacterial virulence factors was conducted by PCR test and one biopsy from each case was used for measuring IL-33 mRNA expression rate by real-time PCR.

**Histological examination**

Gastric biopsy specimens were merged in 10% buffered formalin and stained with hematoxylin and eosin (H&E) to grade gastritis and with giemsa for *H. pylori* detection. The severity of gastritis was graded from 1-3 (Mild, Moderate, Severe) based on the degree of immune cells infiltration, polymor-phonuclear leukocyte (PMN) and mononuclear cell (MNC) infiltration, and dysmorphic according to the updated Sydney system (15).

**Molecular characterization of** ***H. pylori***

Genomic DNA from all samples was extracted by Biospin tissue genomic DNA extraction kit (BioFlux, Japan) PCR. Specific primers for *H. pylori* and its virulence factors PCR test were: 16S rRNA, forward: 5'-CTGGAGAGACTAAGCCCTC C-3' and reverse: 5'-ATTACTGACGCTGATTGTG C-3', glmM (ureA), forward: 5'-AAGCTTTTAG GGGTGTTAGGGGTTT-3' and reverse: 5'-AAGC TTACTTTCTAACACTAACGC-3' (housekeeping and specific genes, respectively), cagA, forward: 5'-ATGACTAACGAAACTATTGATC-3' and reverse :5'-CAGGATTTTTGATCGCTTTATT-3', babA2, forward:5'- CCAAACGAAACAAAAAGCGT - 3' and reverse: 5'-GCTTGTGTAAA AGCCGTCGT-3' . For cagA, babA_2_ gene evaluation, the PCR program contained 35 cycles of denaturation (94 °C for 30 s), annealing (56 °C for 30 s, extension at 72 °C for 30 s), and one final extension (72 °C for 5 min) (16, 17).

**Analysis of IL-33 mRNA expression in the gastric mucosa samples by real-time PCR**

Total RNA was extracted from gastric biopsy samples by total RNA extraction biozol (bioflux, Japan). An aliquot containing 0.1 mg of total RNA was used for the reverse transcription (RT) reaction, according to the manufacturer’s instructions first-strand cDNA synthesis system (Takara, Japan). The sequences of oligonucleotide primers and probe designed by Oligo.7 software for β-actin and IL-33 are: β-actin, forward 5'-AGCCTCGCCTTTGC-CGA-3' and reverse 5' -CTGGTGCCTGGGGCG-3' and probe FAM 5'-CCGCCGCCCGTCCACACC-CGCC-3' TAMRA, for IL-33, forward 5'-TGGAGGATGAAAGTTATGAG-3', reverse 5'-TCAGGGTTACCATTAACATC-3', probe FAM 5'-TACCATCAACACCGTCACCTGATTCA-3' TAMRA. The assessment of IL-33 mRNA levels was performed using a Rotor-Gene 3000 (Corbett, Australia). real-time PCR reactions were done in a total volume of 25 µl containing 12.5 µl of 2x Rotor-Gene Probe PCR Master Mix (Qiagen, Germany), 3 µl of synthesized cDNA solution, 500 nM of each forward & reverse primer and 250 nM of the TaqMan probe. Amplification process included a primary warming step (94 °C for 10 min), denaturation step (94 °C for 15 s) and an annealing/extension step (60 °C for 60 s). Relative quantification of cytokine mRNA to β-actin mRNA was determined using the 2^-∆^Ct method (18). Each assay was done in duplicate.

Statistical analyzes

In statistics, normality tests were used to determine whether a data set is well-modeled by a normal distribution or not. Cytokine expression is presented as means and differences between infected and uninfected groups were analyzed using the Mann-Whitney test. P values<0.05 were considered as significant.

## Results

**Genotype analysis**

Table 1 gives an overview of the frequency distribution of the cagA, babA_2_ status. The babA_2_ gene was found in 49.5% of the *H. pylori* positive biopsies (Table 1). The cagA gene was detected in 68.3% (69 biopsies) of *H. pylori* positive specimens. The frequency of virulence factors in *H. pylori* positive biopsies is shown in table 2.

**Table 1:** Frequency of the cagA and the babA_2_ in studied patients

**Genotype**	**Frequency** N **%**
**cagA** Positive Negative	69 68.3 32 31.7
**babA**_2_ positive negative	50 49.5 51 50.5

**Table 2:** Frequency of virulence factors in *H. pylori* positive biopsies

** Genotype**		** Frequency**	
cagA	babA_2_	N	(%)
+	+	43	62.3
+	-	26	37.7
-	+	7	21.9
-	-	25	78.1

**Fig. 1:**
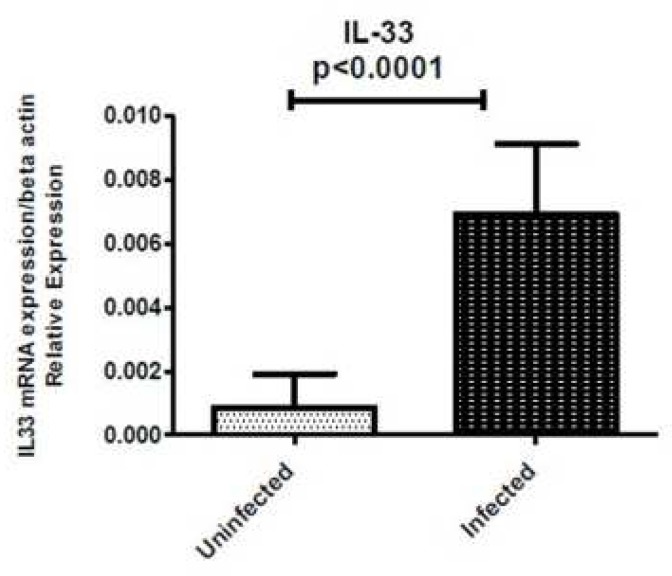
Mucosal IL-33 mRNA expression level in gastritis patients. 79 *H. pylori* infected and 51 *H. pylori* non-infected patients with gastritis were analyzed for IL-33 expression by real-time *PCR*. Levels are normalized to β-actin

**Elevated IL-33 expression in** ***H. pylori*** **-infected gastric mucosa**

IL-33 mRNA level was detectable in all samples regardless of whether biopsies were taken from *H. pylori*-infected or uninfected patients. IL-33 expression was significantly higher in biopsy specimens of *H. pylori*-infected patients compared with uninfected patients (P<0.0001) (Fig. 1). If the mean *H. pylori*-positive group is 0.0069 and the mean *H. pylori*-negative group is 0.0008 then the difference in the IL-33 mRNA expression in the *H. pylori*-positive compared with the *H. pylori*-negative patients is 0.0069/0.0008 or 8.6 fold.

**Effect of cagA and babA**_2_ **on the mucosal IL-33 mRNA levels in** ***H. pylori*** **-infected patients**

**Fig. 2.**
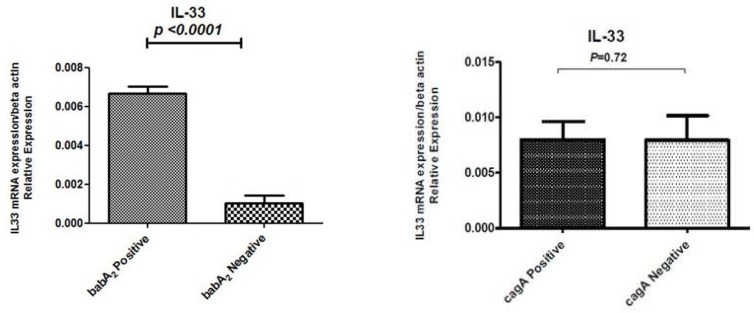
Mucosal IL-33 mRNA expression in *H. pylori* infected patients according to virulence factors. IL-33 mRNA expression level was significantly lower in babA_2_ negative gastritis patients compared with babA_2_ positive patients. There is no significant relationship between IL-33 gene expression and existence of cagA virulence factor

A mucosal IL-33 mRNA level was dependent on virulence factors status. If the mean level of IL-33 in *H. pylori*-cagA positive group is 0.00798 and the mean level of IL-33 in *H. pylori*-cagA negative group is 0.0079, then the difference in the IL-33 mRNA expression in the cagA-positive *H. Pylori* strains in comparison with the cagA-negative *H. pylori* strains biopsies is 0.00798/0.0079 or 1.01 fold. The difference in the IL-33 mRNA expression in the babA2-positive *H. pylori* strains compared with the babA_2_-negative *H. pylori* strains is 0.0067/0.0012 or 5.58 fold (Fig. 2).

**Association between mucosal IL-33 mRNA levels and gastric inflammation classification**

During this study, semi-quantitative methods of scoring according to the updated Sydney system were used. The degree of active inflammation was evaluated and scored as below: 19.8% mild, 24.8% moderate and 19.8% severe, and more than 64% of gastritis patients were in an active stage of *H. pylori* gastritis. In addition the degree of chronic inflammation was assessed and graded as follow: 11.9% mild, 13.9% moderate, 9.9% severe, and more than 35% of gastritis patients were in a chronic phase of *H. pylori* gastritis (Table 3).

**Association between mucosal IL-33 mRNA levels and type of gastritis**

**Fig. 3:**
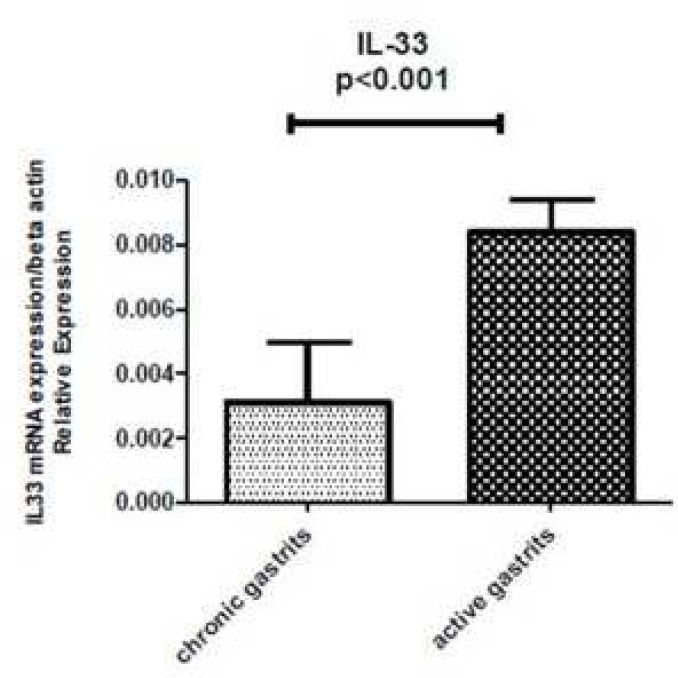
Mucosal IL-33 mRNA levels according to gastritis status. IL-33 mRNA expression level was significantly lower in chronic gastritis patients compared with patients with active gastritis

Figure 3 shows the relationship between the mucosal IL-33 mRNA expression and chronic inflammation (mononuclear cell infiltration) scores. There was a significant correlation between the mucosal IL-33 mRNA expression and the active inflammation scores (P<0.001). If the mean of IL-33 mRNA level in chronic gastritis group is 0.0031 and the mean of IL-33 mRNA level in active gastritis group is 0.0083 then the difference in the IL-33 mRNA expression in the active gastritis compared with the chronic gastritis biopsies is 0.0083/0.0031 or 2.67 fold (Fig. 3).

**Table. 3:** *H.pylori* gastritis status according to updated Sydney classification

**Description**	**Mild** **N (%)**	**Moderate** **N (%)**	**Severe** **N (%)**	**Total** **N (%)**
Active	14 (19.8)	18 (24.8)	14 (19.8)	46 (64.4)
Chronic (no activity)	8 (11.6)	10 (14)	7 (10)	25 (35.6)
*H.pylori* positive	all	all	all	all
Man/woman	10/12	14/14	9/12	33/38
Total	22 (30.99)	28 (39.44)	21 (29.57)	71 (100)

## Discussion

*H. pylori* infection may remain in the host for a long time and *H. pylori* pathogenesis is related to its virulence factors including cagA and babA. The cagA positivity in Iranian isolates varies between 44% to 91% in different reports. In the present study, 68.3% of the patients were infected with cagA- positive strains, similar to other Iranian reports (1, 19, 20). The babA positivity in Iranian isolates has been reported to vary from 40.6% to 94% in different studies. In the present study, 49.5% of the patients were infected with babA-positive strains, similar to other Iranian reports (1, 21, 22).The IL-1 family of cytokines has an established role in immune regulation and inflammatory processes. IL-33 has been observed to supply protection during gastric infection and may be an important mediator of the immune functions after damage or infection in epithelial cells (23, 24). IL-33 has been reported to reduce colonization and pathological effects in gastrointestinal infection (25). Recently several researches suggested that IL-33 may be involved in tumorogenesis and development of vascular diseases (26). In the present study, IL-33 mRNA expression was significantly higher in *H. pylori* infected patients than non-infected patients. Similar to our results, in another study in 2015, IL-33 mRNA expression increased with bacterial infection in stomach (27). However, in another study in 2015, IL-33 mRNA expression was reduced in chronic infection by *H. pylori* (14). In the present study, IL-33 mRNA expression was significantly high in babA_2_ positive *H. Pylori* infected patients as well as active gastritis patients. To the best of our knowledge, this report is the first study considering the association between IL-33 mRNA expression and virulence factors. Like our results, another study in 2015 showed that IL-33 mRNA expression was increased in active gastritis biopsies more than chronic gastritis samples (14). The expression levels of other cytokines from IL-1 family such as IL-18 was higher in *H. pylori*-positive patients and cagA+ infected patients compared with *H. pylori*-negative patients and cagA negative infected patients(28). The results of a study in 2013, showed that there was no association between virulence factors and IL-18 mRNA expression (29). Another study showed that serum levels of IL-33 were significantly higher in patients with gastric cancer than healthy people, suggesting that serum IL-33 levels may have a closer correlation with gastric cancer than IL-18 levels. In other words, elevated serum IL-33 level was found to be an independent prognostic indicator (30). Stimulation through both Toll-Like receptors (TLRs) and Nod-Like receptors (NLRs) are all required for processing and release of IL-18 and IL-33 from normal monocytes (31). Similarly, in another study, elevated IL-33 mRNA expression was detected only in lipopolysaccharide (LPS)- activated monocytes (5). LPSs from Escherichia coli stimulate host immune cells via TLR4 and TLR2 and enhance IL-33 gene expression level in monocytes (32-34). Some data suggest that engagement of both TLR2 and TLR4 pathways stimulates IL-33 indicating a potential role for this cytokine in immune responses to diverse pathogens and pathogen associated microbial patterns (PAMPs) (35).

Our data suggest that reduction in IL-33 mRNA expression level may be a biomarker for predicting the prognosis of gastritis, especially in chronic gastritis which is an initiation for gastric ulcer. Prospective studies in a larger population and more virulence factors should be carried out to confirm the findings.

## Conflict of interest

The authors declared no conflict of interests.
